# Locked in and stressed out: COVID-19 and video-telemedicine in community perinatal mental health services

**DOI:** 10.1192/j.eurpsy.2021.1792

**Published:** 2021-08-13

**Authors:** M. Turki, M. Miele

**Affiliations:** Cnwl Perinatal Mental Health Services, St Mary’s Hospital, London, United Kingdom

**Keywords:** COVID-19, telemedicine, Perinatal Psychiatry, psychiatry

## Abstract

**Introduction:**

The COVID-19 viral pandemic has taken the world by surprise. The pandemic has caused a great impact on the mental health and wellbeing of pregnant women with mental health difficulties. Healthcare providers veered towards video-telemedicine to safely and swiftly provide services to its users.

**Objectives:**

To determine impact of Video-telemedicine on: 1. Access to Care 2. Ease of Use 3. Quality of Care 4. Difficulties of Use 5. Future Prospects of Video Telemedicine

**Methods:**

We have decided to conduct a targeted survey to 100 pregnant women who are known to Perinatal Mental Health services to assess the new methods of contact that the viral pandemic has enforced upon healthcare providers.

**Results:**

Pre-pandemic: video-telemedicine was ranked as least preferred Post-pandemic it is ranked as second favourite. 70.4% of responders have confirmed that video-telemedicine significantly facilitated access to care. 23.3% of responders insisted video-telemedicine made the service better. 50% of responders thought it was much easier to use video options to access their care services needs 95.4% of responders felt that video-telemedicine alternatives should remain post-pandemic

**Conclusions:**

Video-telemedicine options have significantly improved the access and quality of services provided by Community Health Services to pregnant women during the pandemic. Video options can also make it easier to reach critical care without negatively affecting the quality of wholistic care provided, in fact, it can sometimes improve it. It is important that the psychiatric field learn from this pandemic and implement these services permanently. Bigger and wider studies need to be done in the future to support these conclusions.

**Disclosure:**

No significant relationships.

